# Pediatric Pain Management and Sedation

**DOI:** 10.1155/2010/454731

**Published:** 2010-12-26

**Authors:** Savithiri Ratnapalan, Keira P. Mason, Sharon E. Mace

**Affiliations:** ^1^Division of Emergency Medicine and Clinical Pharmacology and Toxicology, The Hospital for Sick Children, University of Toronto, 555 University Avenue, Toronto, ON, Canada M1G 1X8; ^2^Department of Paediatrics and Dalla Lana School of Public Health, University of Toronto, Toronto, ON, Canada M5T 3M7; ^3^Children's Hospital Boston and Harvard Medical School, Boston, MA 02 115, USA; ^4^Cleveland Clinic Lerner College of Medicine of Case Western Reserve, Cleveland, OH 44195, USA

Our ability to provide analgesia and sedation for children has evolved over the past several years. We have progressed from papoose boards to oral sucrose solutions to soothe babies during procedures. Many procedures that were traditionally performed in the operating room are being performed in remote settings: inpatient wards, satellite units, and emergency rooms. The delivery of pediatric sedation is no longer restricted to a limited group of specialists, but instead is delivered by specialists, and physicians as well as nonphysicians, in the field of anesthesia, hospital medicine, pediatrics, intensive care medicine, dental medicine, emergency medicine, and radiology. Some sedatives and analgesics have been introduced to market within the past decade whereas others, still in use, have existed for over a century. 

The ability of infants to recognize pain was initially underappreciated. Clinical and bench research, however, have sensitized us to the newborn's capacity to feel pain and has, subsequently, laid the groundwork for ongoing research into the pathophysiology of pain and clinical tools for proper assessment [1, 2]. Acute pain management options for children continue to evolve, encompassing all routes of delivery: oral, rectal, topical, subcutaneous, mucosal, intramuscular, parenteral and recently intranasal. Some of the recent introductions of this century include our appreciation of the analgesic and sedative benefits of oral sucrose in newborns and the use of alternative delivery routes, such as intranasal fentanyl for analgesia [3, 4]. There has also been continued interest comparing the benefits of nonsteroidal anti-inflammatory medications to narcotics [5]. Despite the advances in our knowledge and application of analgesics, patient safety continues to be a concern, particularly as unexpected adverse events, a morphine overdose in breast milk of a mother taking codeine for example, continue to occur [6]. 

 Analgesia and sedation practices are not uniform; guidelines, policies, and protocols differ among professional organizations, provider groups, countries, institutions and among providers within the same institution. The inability to reach a consensus on safe practice and appropriate guidelines threatens our ability to provide safe, consistent care and fuels debate and malcontent amongst and between some specialties.

The magnitude of human and financial cost of jeopardizing patient safety in sedation is large and adverse outcomes should be rare. The numerical value of rare should not be a percentage; for example, a 99.9% probability of having a given outcome or 0.1% (1 in 1000) probability of a serious adverse outcome as a result of sedation is not acceptable. An acceptable aim for pediatric sedation should be “six sigma” which will reduce adverse outcome to 3-4 errors per a million incidents [7]. 

Ensuring that the practice of pediatric analgesia and sedation follows the same rigorous safety monitoring at all times by all providers, and in any setting across the world is a common responsibility shared by healthcare providers caring for children. 

*Savithiri Ratnapalan* *Savithiri Ratnapalan* *Keira Mason* *Keira Mason* *Sharon E. Mace* *Sharon E. Mace*

## References

[1] Fitzgerald M, Beggs S (2001). The neurobiology of pain: developmental aspects. *Neuroscientist*.

[2] Stevens BJ, Johnston CC, Horton L (1993). Multidimensional pain assessment in premature neonates: a pilot study. *Journal of Obstetric, Gynecologic, and Neonatal Nursing*.

[3] Stevens B, Yamada J, Ohlsson A (2004). Sucrose for analgesia in newborn infants undergoing painful procedures. *Cochrane Database of Systematic Reviews*.

[4] Borland M, Jacobs I, King B, O'Brien D (2007). A randomized controlled trial comparing intranasal fentanyl to intravenous morphine for managing acute pain in children in the emergency department. *Annals of Emergency Medicine*.

[5] Drendel AL, Gorelick MH, Weisman SJ, Lyon R, Brousseau DC, Kim MK (2009). A randomized clinical trial of ibuprofen versus acetaminophen with codeine for acute pediatric arm fracture pain. *Annals of Emergency Medicine*.

[6] Koren G, Cairns J, Chitayat D, Gaedigk A, Leeder SJ (2006). Pharmacogenetics of morphine poisoning in a breastfed neonate of a codeine-prescribed mother. *Lancet*.

[7] Pande PS, Holpp L (2002). *What is Six Sigma?*.

